# Impact of compensation and willingness to keep same career path on burnout among long-term care workers in Japan

**DOI:** 10.1186/s12960-023-00845-1

**Published:** 2023-08-17

**Authors:** Bum Jung Kim, Clara Jung Won Choi

**Affiliations:** 1 Department of Social Welfare, Chung-Ang University, Seoul, South Korea; 2 Center for Policy Research on Aging, UCLA, Los Angeles, United States of America; 3 Policy Research Division, Korea National Council on Social Welfare, Seoul, South Korea

**Keywords:** Compensation, Willingness to keep same career path, Burnout, Long-term care worker, Japan

## Abstract

**Background:**

This study examined the relationships between compensation, willingness to keep same career path, and burnout among long-term care workers in Japan.

**Methods:**

Data were collected from 319 care workers at long-term care facilities in Japan. The study variables included data on demographics, compensation, willingness to keep same career path, and burnout.

**Results:**

The study found that compensation and willingness to keep same career were significantly negatively associated with burnout levels among long-term care workers in Japan. Long-term care workers with high compensation levels were found to be more likely to have low burnout levels. In addition, care workers who expressed an intention to keep same career path were likely to have low burnout levels. In addition, compensation is associated with burnout as the mediating role of willingness to keep same career.

**Conclusions:**

These results highlight the importance of implementing policies and measures that reduce the risk of burnout among care workers to improve the quality of care. Strategies for improving working conditions include increasing wages, increasing compensation for experienced care workers, and reorganizing benefits.

## Introduction

Japan’s population has been aging rapidly over the past few decades due to decreasing birth rates and increasing life expectancy. In 2016, the life expectancy of Japanese men and women was estimated to be 81.0 and 87.1 years, respectively [1]. Japan has the world’s largest aging population. In 2017, 27.7% of Japanese residents were older than 65 [2]. Japan’s government has formulated policies to respond to the aging population's needs and prevent the need for long-term care. Families feel considerable pressure to assume the burden of caring for older adults, many of whose children are in their 60s, and their mean age is increasing; they often require care themselves [3]. Moreover, younger people are increasingly working outside their hometowns, and housing has not improved to ensure adequate living space for older adults. The informal caregiving system is approaching crisis due to changes in family structure from extended large family to nuclear family, with fewer children and women and changing attitudes toward caregiving [4].

Long-term care was introduced in 2000 under the national insurance policy to respond to these problems [5]. Long-term care was established for three primary reasons. The first and main reason was to remove the burden of informal caregiving. The second was to shift the cost burden through insurance. The third was to combine medical care and social welfare services for older adults. The number of insured older adults increased 1.6 times from 2000 to 2015 (20 million to 33 million; [1]). Long-term care provided through universal social insurance has advanced healthcare in Japan [6]. As demand for long-term care grows, the capabilities of long-term caregivers become increasingly important. Care worker burnout affects patients. When burnout increases, motivation for one's job is also weakened and job satisfaction is lowered. As one's own stress cannot be managed, care and interest in others are lowered, and thus care for clients is likely to be neglected [7]. Care workers with a high degree of burnout are less likely to provide high-quality care, and burnout can reduce their abilities and increase the risk of medical errors [8]. Therefore, care worker burnout may also be related to clinical outcomes, such as patient perceptions of the quality of their care. However, this has not been examined empirically.

### Burnout

Burnout is the depletion of energy and emotional resources [9, 10]. The condition was first reported among workers in high-income nations and has been studied almost exclusively in the developed world. Prior studies indicate that burnout has three major components: emotional exhaustion, depersonalization, and reduced personal accomplishment [11, 12]. Burnout comprises either physical or emotional exhaustion, usually caused by work stress. Human services experts are the most commonly affected workers [13]. Burnout is also viewed as a problematic mental strain caused by continuous interaction with people in need, leading to client depersonalization.

Although burnout is difficult to comprehensively measure and quantify, recent findings suggest that it may erode professionalism, influence quality of care, increase the risk of medical errors, and promote early retirement [14–17]. Burnout also appears to have adverse personal consequences for caregivers, including contributing to broken relationships, problematic alcohol use, and suicidal ideation [16, 18, 19]. Since burnout may be understood as a long-term effect of work, it can indicate perceptions of work life quality [20]. Significant burnout can occur when employee benefits are cut, corporate ownership is changed, frequent overtime is required, or the workforce is reduced [21]. The long-term care workforce in the study includes not only direct care workers and social workers, but also nurses, nurse assistants, occupational therapists, and physical therapists. In addition, long-term care insurance in Japan is controlled by the public sector, but the actual service provision is not limited to the public, because many private and non-profit organizations are also involved in service provision.

### Literature review

#### Compensation and burnout

The Selective Optimization with Compensation (SOC) model proposes strategies for optimal personal resources allocation, maintenance, and function enhancement amid resource losses [22]. The model was developed to explain how individuals with diminishing resources can cope with the effects of aging, such as illness and physical deterioration. In the model, selected goals are prioritized and pursued through goal-relevant means, using compensatory methods to maintain goal attainment when previously employed resources are no longer available or are blocked.

This model has been applied to explain optimal functioning among individuals experiencing burnout [23]. The combined use of selection, optimization, and compensation strategies was found to buffer the unfavorable effects of disengagement on supervisor ratings of task performance and adaptivity to organizational change. Compensation was found to be the most effective strategy for buffering the negative effects of burnout on such ratings. Individuals may compensate for burnout using various external resources, such as help from others or technology, by increasing their efforts, or learning new skills [24]. Contrariwise, selection was found to exacerbate the negative relationship between exhaustion and adaptivity to organizational change, but not task performance. Burnout was significantly high when employee compensation was reduced [21]. According to the literature, burnout of long-term care workers is very high and they are exposed to stress and mental fatigue. In addition, the salary level of long-term care workers is low. Therefore, as mentioned in the SOC model, when employee compensation is reduced and the burnout level is low, the high burnout level of long-term care workers receiving low salaries is understandable, and thus the burnout can be reduced by increasing the compensation [21, 22].

#### The negative association between willingness to keep same career path and burnout

Several studies have found a significantly negative association between willingness to keep same career path and burnout among long-term care workers, emphasizing the need to examine the human service profession’s long-term impact on worker burnout. Korean research on social workers found that the shorter social workers’ continuous work tenure, the greater their burnout [25]. Moreover, length of continuous stay in current employment is associated with less burnout [26]. Furthermore, most human service workers continue to work despite their risk of burnout, increasing the evidence of a relationship between work discontinuation and burnout [27]. People also develop attitudinal affections toward their work, which turn into high commitment and satisfaction [28, 29]. Work discontinuation may threaten such primary affections [30]. It can also decrease productivity and increase absenteeism [31].

#### Willingness to keep same career path as a mediator between compensation and burnout

Several studies have suggested that compensation is positively associated with willingness to keep same career path [32, 33], which appears to be a powerful predictor of burnout among care workers [28–31]. Compensation strategies comprise a set of personal behavioral resources that positively predict favorable work outcomes such as willingness to keep same career path [34, 35]. This willingness should be lower if the compensation strategy does not facilitate worker motivation [36]. Therefore, compensation is likely to be positively associated with willingness to keep same career path.

Studies have demonstrated a positive association between willingness to keep same career path and burnout [28, 30, 31]. The stronger the association between them, the greater the former’s effect on worker burnout. Recent studies have shown supportive results [25, 26, 28, 30, 31]. Although numerous studies investigate the relationships between compensation, willingness to keep same career path, and burnout, research on long-term care workers has rarely been studied. In particular, there is a lack of studies examining the role of mediating factors on willingness to keep same career path in relation to compensation and burnout. Since identifying the relations among these factors is crucial to draw implications promoting the overall quality of long-term care services provided by care workers, it is necessary to verify the associations and relationships.

### Purpose of the study and conceptual model

Several studies have verified the negative relationship between compensation and willingness to keep same career path and burnout, but none has examined the mediating role of willingness to keep same career path. Few studies have investigated how work is affected by compensation and how this affects care worker burnout [21, 23, 24]. Therefore, this study aims to examine whether willingness to keep same career path has a mediating role the relationship between compensation and burnout among long-term care workers using path analysis. The study empirically investigates how important willingness to keep same career path is to care workers by evaluating the path and relationship between the variables.

**Fig. 1:**
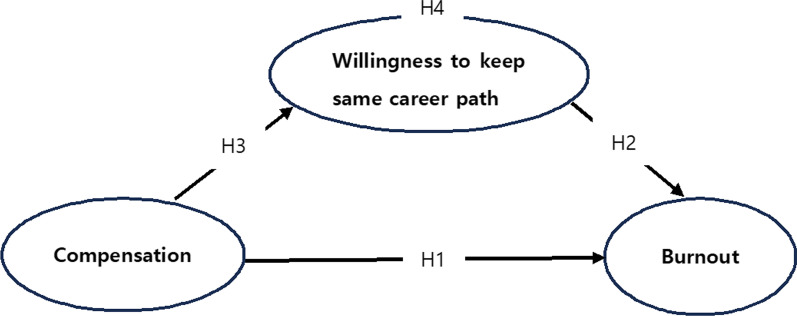
Conceptual model

The hypotheses (Fig. 1) for the current study are as follows.

**H1.** Compensation is negatively associated with burnout among long-term care workers in Japan.

**H2.** Willingness to keep same career path is negatively correlated with burnout.

**H3.** Compensation is positively related to willingness to keep same career path.

**H4.** Willingness to keep same career path has a mediating role in the relationship between compensation and burnout.

## Methods

### Data collection

Survey data were collected from 360 long-term care workers at facilities so defined under Article 210-5 of Japan's Elderly Welfare Act. Data were collected from December 2016 to January 2017 using convenience sampling. The research team contacted acquaintances working at long-term care facilities in Japan to find out whether a survey was possible, and found that institutions in several prefectures showed interest. As a result, the research team collected data from 15 organizations in Osaka, Kobe, Kyushu, Nagasaki, Hokkaido, Tokyo, and Niigata, Japan. After a research cooperation agreement was signed with program directors of each institution, survey participants were identified; notices were retrieved; and self-administered questionnaires were distributed and filled out. The final sample consisted of 319 long-term care workers out of 360, excluding missing data that did not meet the study criteria.

Participation in the study was voluntary, and confidentiality was ensured. Before recruitment, the survey team explained the survey’s purpose and procedure to prospective participants. Prior written consent was obtained from all participants, and Kwansei Gakuin University institutional review board approval was acquired. The survey consisted of standardized Japanese surveys [21, 37] created using translation and coordination techniques. This process was carried out by the first bilingual translator, who translated the English version of the survey tool into Japanese; and the second bilingual translator, who back-translated the tool from Japanese into English. Subsequently, two translators translated each interview for confirmation.

### Measures

*Burnout* Burnout level was measured using the Maslach Burnout Inventory (MBI), developed by Maslach and Jackson [37]. The 17-item scale has three dimensions: emotional exhaustion, depersonalization, and attainment of personal fulfillment. Responses are based on a five-point Likert scale (“never,” “rarely,” “every once in a while,” “sometimes,” “almost always”). A higher score indicates higher burnout risk. The internal reliability of the MBI was 0.87.

*Compensation system* Four questions investigated the compensation system of long-term care workers’ employers: one on promotion opportunity, one on salary adequacy, and two on the welfare system. Previous studies set these factors as sub-variables of the job environment, and some studies used organizational characteristics. However, this study classified them as part of the compensation system [21]. Each question was reverse-scored, with responses ranging from 1 (“very strong”) to 5 (“very weak”), using a five-point Likert scale. A higher score indicated a stronger compensation support system. The internal reliability of the compensation system was 0.91.

*Willingness to keep same career path* We measured degrees of willingness to keep same career path by asking, “Are you willing to keep on working as a care worker in the future?” Response options were “yes” or “no.”

*Background information* Background information was collected for each participant. Sociodemographic variables included age (in years), gender (1 = woman), marital status (1 = married), education (continuous variable), and income (continuous variable).

### Data analysis

Descriptive statistics were used to describe the general characteristics of the sample, and Pearson correlation coefficients were used to examine bivariate associations among the variables. We also investigated intercorrelations and variance inflation factors to determine multicollinearity. Multiple regression analysis was conducted to determine how the key independent variables affected burnout while controlling the sociodemographic variables, such as age, gender, marital status, education, and income. To test the research hypotheses, we verified the research model via path analysis using structural equation modeling software (STATA 13.0, StataCorp LLC, College Station, Texas, USA). The SEM program is designed to estimates path coefficients for a sample with incomplete data using maximum likelihood (ML) algorithm (missing data replacement) [38]. However, when the original total sample (*n* = 360) with missing data was used for path analysis, SEM provided estimates with relatively insufficient general fit of the model. Therefore, pairwise deletion of missing data was carried out as an optimal alternative. This left 319 cases for the analyses of this study. The SEM program estimates path models using variance–covariance matrices. For individual paths, maximum likelihood estimate (MLE) was tested for statistical significance using the critical ratio (values [1.96 indicate statistical significance at the *p*<0.05 level) [39].

## Results

### Sample characteristics

Table 1 presents descriptive data on the participants’ sociodemographic characteristics. The participants’ average age was 54.24 years (standard deviation [SD] = 6.30), ranging from 26 to 70 years. Approximately 94% of the sample were women, and 92% were married. On average, they earned $2520.90 (SD = 1058.97) per month. Approximately 60% of the participants had graduated from high school, and approximately 20% had graduated from college. Compensation and benefits ranged from 0 to 15, with an average of 7.79 (SD = 2.55). Of the respondents, 88% indicated a wish to continue as long-term care workers, and 11% indicated that they did not wish to continue. Burnout scores ranged from 0 to 49, with an average of 19.81 (SD = 8.93).

**Table 1:** Descriptive characteristics of the study sample (*N* = 319)

Variable	*n*	%
Age (in years)		
Range	26–70	
Mean (SD)	54 (6)	
Gender^a^		
Woman	300	94
Man	19	6
Marital status^b^		
Not married	25	8
Married	294	92
Education (in years)		
Elementary	9	2
Middle	53	16
High	190	60
College	39	12
Graduate	28	9
Income (monthly in dollars)		
Range	2200–3650	
Mean (SD)	2520.90 (1058)	
Compensation		
Range	0–15	
Mean (SD)	8 (2)	
Willingness to keep same career path		
Yes	282	88.96
No	37	11.04
Burnout		
Range	0–49	
Mean (SD)	20 (9)	

*SD*  standard deviation

^a^Gender: woman = 1, man = 0

^b^Marital status: single = 0, married = 1

### Variable correlations

Table 2 shows the imputed correlations between the variables in the predictive model. No correlation coefficient value was above 0.60, indicating a lack of multicollinearity between the variables [40]. A significantly positive correlation was observed between compensation and willingness to keep same career path, and increased reward and benefit levels were associated with a higher likelihood of continuing work. There was also a significantly negative correlation between compensation and burnout and a significantly negative correlation between willingness to keep same career path and burnout. Thus, increased compensation levels were associated with lower burnout levels, and lower work sustainability was related to higher burnout levels.

**Table 2:** Correlations among study variables (*r* scores, *N* = 319)

	1	2	3	4	5	6	7
1. Age							
2. Gender^a^	0.15**						
3. Marital status^b^	0.39**	0.12*					
4. Education	− 0.31**	− 0.28**	− 0.14*				
5. Income	− 0.14**	− 0.06	0.03	0.29**			
6. Compensation	0.03	0.05	− 0.00	0.02	0.21**		
7. Willingness to keep same career path	− 0.10	− 0.16**	− 0.00	0.04	0.01	− 0.27**	
8. Burnout	− 0.14**	− 0.07	− 0.06	0.13*	− 0.08	− 0.32**	0.34**

^a^Gender: woman = 1, man = 0

^b^Marital status: single = 0, married = 1

* *p* < 0.05 ** *p* < 0.01

### Regression model with sociodemographic variables

Before the path analysis, multiple regression analyses were conducted to determine the relative contributions of compensation and willingness to keep same career path in the prediction of burnout while controlling for sociodemographic variables (see Table 3). Of the sociodemographic variables, only education level contributed significantly to burnout. However, the two main independent variables (compensation [*β* = − 0.83, *p* < 0.01]) and willingness to keep same career path [*β* = 7.71, *p* < 0.01]) contributed to burnout significantly.

**Table 3:** Multiple regression models of burnout among care workers

Step	*B*	*T*	SE
1			
Age	− 0.10	− 10.30	0.08
Gender	1.15	0.57	2.03
Marital status	− 0.30	− 0.17	1.85
Education	1.30*	2.41	0.53
Income	− 0.04	− 1.60	0.02
2			
Compensation	− 0.83**	− 4.41	0.18
Willingness to keep same career path	7.71**	5.03	1.53

*p < 0.05 **p < 0.01

### Path analysis

The regression model showed that the two main independent variables (compensation and willingness to keep same career path) contributed significantly to burnout; however, limited measurability is a problem: only the direct influence of the independent variable is visible. Therefore, we tested a comprehensive analysis model of burnout via pathway analysis, including direct and indirect effects between the key variables.

As Table 4 shows, compensation was significantly negatively associated with willingness to keep same career path. Moreover, willingness to keep same career path was significantly positively associated with burnout, and compensation was directly and negatively associated with burnout. Indirect effects involve chains of straight arrows called “compounded paths,” wherein the path along the arrow is always forward [41]. As Table 5 shows, the results of the path analysis indicated an indirect pathway of interest. Compensation was negatively associated with willingness to keep same career path, which led to reduced burnout levels. Comparing the sizes of the total effect (see Table 5) reveals that the willingness to keep same career path was strongly associated with burnout (*β* = 7.81, *p* < 0.01), indicating that willingness to keep same career path was a strong risk factor for burnout in the model. Furthermore, compensation (*β* = − 1.15, *p* < 0.01) was another crucial risk factor for burnout.

**Table 4:** Path analysis results

	Unstandardized estimates	Standardized estimates	*Z* value
Willingness to keep same career path ← Compensation	0.00	− 0.03	− 5.07**
Burnout ← Willingness to keep same career path	1.51	7.81	5.17**
Burnout ← Compensation	0.18	− 0.89	− 4.82*

*p < 0.05 **p < 0.01

**Table 5:** Standardized direct, indirect, and total effects predicting life satisfaction

	Direct effects	Indirect effects	Total effects
Willingness to keep same career path ← Compensation	− 0.03**		− 0.03**
Burnout ← Willingness to keep same career path	7.81**		7.81**
Burnout ← Compensation	− 0.89*	− 0.26**	− 1.15**

*p < 0.05 **p < 0.01

## Discussion

This study investigated the relationships between compensation, willingness to keep same career path, and burnout for 319 long-term care workers in Japan. The study’s main implications are as follows.

This study found that compensation is significantly negatively associated with burnout levels among long-term care workers. Thus, long-term care workers with high compensation levels are more likely to have low burnout levels. This result is consistent with previous findings [21, 23, 24].

Long-term care workers perform non-regular contract work for hourly wages; in fact, more than 40% are non-regular workers. Because their job status is low, their wages are also low, and the overall compensation system is inadequate. Strategies for improving working conditions and the compensation system are proposed below.

First, wages must be increased. A significant amount of the non-regular work based on hourly wages should be transitioned to regular work paid monthly. Moreover, hourly wages must be increased. It is necessary to increase professionalism and recognize workers as upholders of professional values. For example, specialization in dementia care could be enhanced through education, increasing the capacity to care for patients with dementia and strengthening areas, such as exercise and rehabilitation. If wages do not rise, the treatment of long-term care workers receiving low wages is not improved and their pride in their jobs are weakened as they do not receive adequate compensation, further triggering the turnover of amongst capable workers, resulting in lowering service quality.

Second, higher compensation should be offered for more experienced care workers. Experience does not always make the job easier or the worker more competent. In general, however, work experience does increase proficiency and competence. Introducing an incentive system should thus be considered to encourage worker longevity. Incentives should be provided to long-term care workers with specialized skills and knowledge to support the work of more experienced care workers and strengthen the overall compensation system. Introducing a promotion system should also be considered. Promoting workers recognizes achievement and increases their salaries, which should increase work satisfaction.

Third, the benefits for long-term care workers should be strengthened. Expanding benefits through subsidies for meals and transportation and health check-ups to improve workers’ health management should increase worker satisfaction and institutional credibility. High-quality, effective services can be provided only when service providers are healthy. The compensation system should also be strengthened by providing bonuses, and welfare allowances.

In fact, it is not clear whether all three policy implications are necessary to lead adequate compensation or if one is more important than the others. However, it is important to recognize that these implications could be tried out to see if these aspects actually influence the behaviors of long-term care workers in the way they perceive it might. Therefore, further research to identify the types of compensation and the most effective combination possible is needed from the implementation perspective.

This study also found a significantly positive relationship between willingness to keep same career path and burnout among long-term care workers. Thus, care workers who intend to keep same career path are likely to have low burnout levels. This finding is consistent with prior results [25, 26, 28, 29].

Long-term care workers have short careers and high turnover rates. It has been reported that many long-term care workers do not use their professional licenses. Studies have found that the compensation system, organizational culture, facility environment, and relationships with clients have important effects on worker turnover [25, 28, 29]. We, therefore, suggest several ways of increasing worker longevity.

First, improving the compensation system is crucial. Second, a flexible organizational culture would reduce job stress and increase years of service. When workers and supervisors have a flexible and smooth relationship, organizational satisfaction is high and the probability of working longer hours increases. Therefore, organizational cultures should feature open communication between supervisors and workers and allow workers to share any grievances or concerns comfortably. Leadership is crucial to creating such a work culture of respect and communication. Moreover, relationships with clients are extremely important. Clients and employees require a culture of mutual respect that provides opportunities for an open dialogue through which they can become acquainted. In addition, increased counseling with the client’s family will enhance the client’s understanding, thus increasing job satisfaction and improving job competency. Finally, we need to create pleasant and comfortable facilities and environments by improving working conditions. Providing clean environments and rest spaces is also important, because long-term care workers perform high-intensity manual labor. Their privacy must also be ensured.

In addition, this study showed that compensation affects willingness to keep same career path, which sequentially affects burnout. These results support the findings of previous studies around the topic [32–35]. These studies have confirmed that compensation and willingness to keep same career path have a strong correlation. In other words, it could be argued that it is necessary to increase the compensation level to enhance the level of willingness to keep same career path.

The present study also found that the level of education had a significantly negative effect on burnout. The field of long-term care is generally known as a field with low wages and high-intensity labor. Long-term care workers usually have a low level of education (approximately 80% of them have a high school diploma or less), and thus many workers with low educational level enter the career path. College graduates and above account only for about 20% of the total long-term care workers, which is considered rather low. In the case of highly educated long-term care workers, they usually join such career path to increase their later-life income after retirement from their primary first job. Originally, in the case of long-term care worker with high educational level, they tend to experience high stress due to low wages and high-intensity physical labor after living a relatively high-income life in a stable job. Therefore, due to amount of work that is involved in long-term care provision and the treatment that does not meet their expectations, increase in mental and physical stress can be evident, which then eventually result in an increase in the level of burnout.

According to the results of the study, gender, marital status, and income did not significantly affect burnout. Such result can be explained by the study sample. 94% out of the study participants is female, 92% is married, and the wage range is not wide and average wages are as low as $2500. In other words, the variation of those variables is not large, which is typical of long-term care workers in Japan. Long-term care workforce is one of the most female-dominated occupations, many are married, and their wages are very low compared to other occupations. Since the variation within the variable is not large, the effect on burnout is not significant.

### Limitations

This study had several limitations. First, causal relationships between the variables could not be determined, because it used a cross-sectional design. Future longitudinal studies should be conducted to examine the causal relationships. Second, the results might lack generalizability, because the study did not include long-term care workers from all regions of Japan. Future research should obtain more representative data by including long-term care workers from a wider geographic area. Third, analyzing the factors influencing willingness to keep same career path would produce a more rigorous research model and more interesting results. Finally, in this study, 'willingness to keep same career path ' is used to refer to whether there is a will to continue working in the future. In Japan's long-term care sector, concepts of tenureship or retirement do not exist, and it is a structure that allows you to continue working if you have your own will. However, since retirement can be considered for various personal reasons (physical health, etc.), it is perceived that there are limitations in interpretation.

## Data Availability

No public availability.
